# Multi-omics profiling reveals sphingolipid metabolism reprogramming of tumor-conditioned MDSCs in cervical cancer

**DOI:** 10.1186/s12885-026-16914-2

**Published:** 2026-09-18

**Authors:** Qiuwen Mai, Chudan Chi, Xiaojun Wang, Yili Chen, Qiaojian Zou, Qianrun Chen, Feitianzhi Zeng, Mengxun Wei, Yanfei Chen, Aiting Wang, Yan Liao, Yuzhou Xiao, Xinjie Li, Qing Yan, Liping Zhan, Hongsuo Wei, Xu Jing, Qiqiao Du, Junxiu Liu

**Affiliations:** 1 Department of Obstetrics and Gynecology, The First Affiliated Hospital of Sun Yat-sen University, Guangzhou City, Guangdong Province P.R. China; 2 Guangdong Provincial Clinical Research Center for Obstetrical and Gynecological Diseases, Guangzhou City, Guangdong Province P.R. China; 3 Department of Obstetrics and Gynecology, Boai Hospital of Zhongshan City, Guangzhou City, Guangdong Province P.R. China; 4 Department of Pathology, Guangdong Women and Children Hospital, Guangzhou City, Guangdong Province P.R. China; 5 Department of Microbiology, Tumor and Cell Biology, Karolinska Institute, Stockholm, Sweden

**Keywords:** Myeloid-derived suppressor cells, Cervical cancer, Sphingolipid metabolism, Multi-omic profiling, Kng1

## Abstract

**Background:**

Myeloid-derived suppressor cells (MDSCs) play a crucial role in the tumor microenvironment (TME) of cervical cancer (CC), yet the mechanisms underlying their reprogramming remain poorly understood.

**Methods:**

To explore the immune microenvironment change in CC, we applied TCGA-CC immune microenvironment infiltration estimation analysis via Timer 2.0 online datasets. To generate tumor-conditioned MDSCs, the culture medium of MDSCs was supplemented with supernatants from the murine CC cell lines U14 and TC1, respectively. CCK8 assays, transwell migration experiments and qPCR were executed to test the proliferation, migration and iNOS expression. We then conducted proteomics and metabolomics analyses of tumor-conditioned MDSCs.

**Results:**

The tumor immune microenvironment analysis identified MDSCs as key components, predicting poor prognosis in CC. Tumor-conditioned MDSCs presented higher proliferation, migration and iNOS expression. Proteomics and metabolomics analyses showed significant changes in lipid metabolism, especially sphingolipid metabolism. Specifically, the Kng1–sphingosine 1-phosphate axis was identified as a central protein–metabolite regulatory node. Gain- and loss-of-function experiments confirmed that KNG1 modulates multiple cellular processes including proliferation, migration, iNOS expression, and sphingosine 1-phosphate production.

**Conclusion:**

Our findings uncover sphingolipid metabolic reprogramming as a key mechanism in MDSCs-mediated immune suppression, and propose the Kng1–sphingosine 1-phosphate network as a potential therapeutic target for CC treatment.

**Supplementary Information:**

The online version contains supplementary material available at https://doi.org/10.1186/s12885-026-16914-2.

## Background

Cervical cancer (CC) is one of the leading causes of female death globally [1]. The CC tumor microenvironment (TME) is a complex ecosystem that plays a critical role in the initiation, progression, metastasis, and therapeutic response of the disease [2]. The immune microenvironment in CC is important, but the immune infiltration environment is still worth further investigation [3]. Numerous studies have evaluated the immune microenvironment, determined immune subsets, constructed an immune cell infiltration scoring system, and screened key immune-related genes [4].

Myeloid-derived suppressor cells (MDSCs), one of the most essential parts of TME in CC, play a significant role in the progression and immune evasion of CC [5]. MDSCs are known to suppress immune responses and protect tumor cells from immune attacks. They contribute to tumor progression via immunological and non-immunological mechanisms, such as inhibiting T cell function, promoting angiogenesis, and pre-metastatic niche formation [6]. The expansion of MDSCs has been observed in both CC patients and tumor-bearing mice, with frequencies positively correlated with tumor burden and negatively with antitumor therapy response and overall survival [7]. Metabolic changes in MDSCs are crucial for their function and survival within the TME [8]. MDSCs adapt to the TME by rapidly and effectively reprogramming their metabolism to maintain immunosuppressive activity and adapt to stressed conditions [9]. This includes controlling the availability of nutrients and oxygen and manipulating receptor signaling events that affect the fate of MDSCs [10]. However, the metabolic reprogramming in MDSCs in CC TME requires further investigation.

The present study identified MDSCs as key components by analyzing the tumor immune microenvironment, predicting a poor prognosis in CC. Proteomics and metabolomics analyses of tumor-conditioned MDSCs in CC showed significant changes in lipid metabolism, especially sphingolipid metabolism. Furthermore, the integrated analysis identified Kng1–sphingosine 1-phosphate as the key protein–metabolite network in CC cell-conditioned MDSCs, providing a detailed mechanism understanding of MDSCs in CC TME.

## Methods

### Timer 2.0 dataset analysis

The TIMER 2.0 database is a comprehensive resource for exploring the landscape of immune infiltrates across various human cancers, revealing the complex interactions within the tumor microenvironment. Utilizing the TIMER 2.0 Dataset, we applied TCGA-CC immune microenvironment infiltration estimation analysis to investigate the CC immune microenvironment.

### Flow cytometry

Single-cell suspensions were washed twice with PBS and stained with Horizon™ Fixable Viability Stain 780 (BD Biosciences) for 15 min at room temperature in the dark. Cells were then blocked with purified anti-mouse CD16/CD32 (clone 2.4G2, BD Biosciences) at 4 °C for 5 min, followed by surface staining with APC-conjugated anti-CD11b and FITC-conjugated anti-Gr-1 antibodies for 30 min at 4 °C. Analysis and sorting of CD11b^+^Gr1^+^ MDSCs were performed using a FACSAria III flow cytometer (BD Biosciences).

### Clinical specimens

Clinical specimens were collected between January 2015 and December 2017 from the First Affiliated Hospital of Sun Yat-sen University, comprising 50 cases of CC paraffin-embedded tissues and 10 normal cervical tissues as controls. Following the Declaration of Helsinki, the study was approved by the Ethics Committee of the First Affiliated Hospital of Sun Yat-sen University. Inclusion criteria for CC patients included: (1) a pathological diagnosis of squamous cell carcinoma, adenocarcinoma, or adenosquamous carcinoma of the cervix. (2) staging from IA2 to IIA2 (FIGO 2009) with patients undergoing radical hysterectomy and lymph node dissection. (3) patients had not received radiotherapy or chemotherapy before surgery. Normal cervical tissues were derived from patients who underwent hysterectomy for non-malignant diseases.

### Tissue immunofluorescence

Immunofluorescence was performed on paraffin-embedded tissue sections. After deparaffinization, rehydration, and antigen retrieval, sections were blocked with 4% normal goat serum. Primary antibodies against CD11b (#21851-1-AP, Proteintech) and CD33 (#17425-1-AP, Proteintech) were applied and incubated overnight at 4 °C. Following washing, sections were incubated with appropriate fluorescent secondary antibodies and counterstained with DAPI. Finally, slides were digitally scanned using a JiangFeng slide scanning system.

### Induction and differentiation of bone marrow cells into MDSCs

The study utilized 6–8-week-old female specific pathogen-free C57BL/6 mice obtained from the Laboratory Animal Center of Sun Yat-sen University (Guangzhou, China). All animals were housed under standard temperature and humidity conditions. For euthanasia, mice were first deeply anesthetized with isoflurane inhalation (induction: 3–4%; maintenance: 1.5–2% in oxygen) until the loss of the pedal withdrawal reflex was confirmed, followed by cervical dislocation. This method was chosen to ensure that animals were unconscious prior to physical euthanasia, minimizing pain and distress in accordance with institutional and international guidelines. Bone marrow cells were aseptically harvested from both femurs and tibias. After erythrocyte lysis and filtration through a 75 μm strainer, the bone marrow cells were uniformly inoculated into 24-well plates at a density of 2 × 10⁶ cells/well in a total volume of 2 mL. Cells were induced to differentiate into MDSCs in RPMI-1640 medium supplemented with 10% fetal bovine serum, 1% penicillin–streptomycin, interleukin-6 (IL-6, 20 ng/mL), and granulocyte–macrophage colony-stimulating factor (GM-CSF, 20 ng/mL). After 3 days of culture under a 5% CO₂ atmosphere at 37 °C, a half-volume media change was conducted by carefully aspirating 1 mL of the old media without disturbing the cell layer and gently adding 1 mL of fresh complete medium containing the same concentrations of IL-6 and GM-CSF. After 5–7 days of total induction, MDSCs were harvested, and the induction efficiency was evaluated by flow cytometry before use in subsequent experiments.

### RNA isolation and qPCR

The SteadyPure Universal RNA Extraction Kit (#AG21017, Accurate Biology) was used to streamline RNA isolation from diverse biological samples. RNA concentration and purity were measured with a NanoDrop 2000 spectrophotometer. Equimolar amounts of RNA were reverse transcribed into cDNA using the Evo M-MLV Reverse Transcriptase PreMix (#AG11706, Accurate Biology). Quantitative PCR was conducted with the SYBR Green Pro Taq HS Premix qPCR Kit (#AG11701, Accurate Biology). Data analysis was performed using the 2^-ΔΔCT method. Primer sequences were listed in Supplementary Table 1.

### Western blotting

Total protein was extracted using radioimmunoprecipitation assay lysis buffer (#P0013C, Beyotime) supplemented with a phosphatase inhibitor cocktail (#P1081, Beyotime) as needed. Proteins were separated by sodium dodecyl sulfate–polyacrylamide gel electrophoresis using a pre-stained protein marker (#WJ103, Epizyme). Subsequently, the proteins were transferred onto polyvinylidene fluoride membranes via the wet transfer method. After blocking, the membranes were incubated with primary and secondary antibodies. Protein bands were visualized using an enhanced chemiluminescence detection system.

### Enzyme-linked immunosorbent assay

Sphingosine 1-phosphate concentrations in cell culture supernatants were quantified using a sandwich ELISA (#MM-44778M2, Meimian). According to the manufacturer’s protocol, samples and standards were incubated, followed by successive incubations with biotinylated detection antibodies and HRP-conjugated streptavidin. After adding tetramethylbenzidine substrate, the reaction was terminated with stop solution, and the optical density was measured at 450 nm.

### Cell culture

The murine cervical carcinoma cell line, U14, along with the HPV-16 E6 and E7 protein-expressing TC-1 cells, were procured from iCell Bioscience Inc., Shanghai. U14 was cultivated in DMEM and TC-1 cells in RPMI-1640, enriched with 10% FBS and fortified with a 1% antibiotic solution comprising penicillin and streptomycin. Both cell lines were maintained in a controlled environment with a 5% CO_2_ atmosphere at a constant temperature of 37 °C.

### Cell proliferation assay (CCK-8)

A total of 2 × 10^4^ MDSCs were seeded in each well of 96-well plates and cultured in a 1:1 mixture of U14-conditioned medium (or TC1-conditioned medium) and fresh complete RPMI-1640 medium (containing 10% FBS and 1% penicillin–streptomycin) under a 5% CO_2_ atmosphere at 37 °C. The Cell Counting Kit-8 (#GK10001, GLPBIO) was utilized to assess cell proliferation. All procedures were conducted following the manufacturer’s instructions. The assessment was performed at 0 h, 24 h, 48 h, and 72 h post-treatment. The absorbance was detected at the time points as indicated at 450 nm.

### Cell transfection

To knock down or overexpress Kng1 in MDSCs, siRNA targeting mouse Kng1 and a plasmid encoding mouse Kng1 were purchased from OBiO (Shanghai, China). MDSCs were induced for 5–7 days as described above, and transfections were performed using Lipofectamine RNAiMAX (for siRNA) and Lipofectamine 3000 (for plasmid) (Invitrogen, USA), respectively, following the manufacturer’s instructions. Transfection efficiency was verified by Western blot analysis. Further functional experiments were conducted 48 h after transfection.

### Transwell migration assays

A 24-well Transwell system with polycarbonate membrane inserts (5.0 μm pore size, #3421, Corning) was utilized for migration assays. Murine CC cell line U14 and TC1 culture supernatants were added into the culture medium of MDSCs to establish the CC-conditioned MDSCs. A total of 1 × 10^6^ MDSCs were inoculated into the upper chamber of each insert. Following incubation, cells on the lower surface of the membrane were fixed with 4% paraformaldehyde for 15 min and stained with 0.1% crystal violet for 30 min. The migration cells were quantified using Image J software.

### Experimental design and statistical rationale

Statistical analyses were performed with SPSS 20.0 statistical software. All values are shown as the mean ± Standard Error of Mean. For 2-group comparisons, a two-sided unpaired Student’s t-test was performed. For multiple comparisons, the one-way ANOVA statistical analysis was applied. *P*-values of *P < 0.05 were considered statistically significant; **P < 0.01 very significant; ***P < 0.001 extremely significant.

## Results

### MDSCs were identified as key components by the tumor immune microenvironment analysis, predicting poor survival in CC

**Fig. 1:**
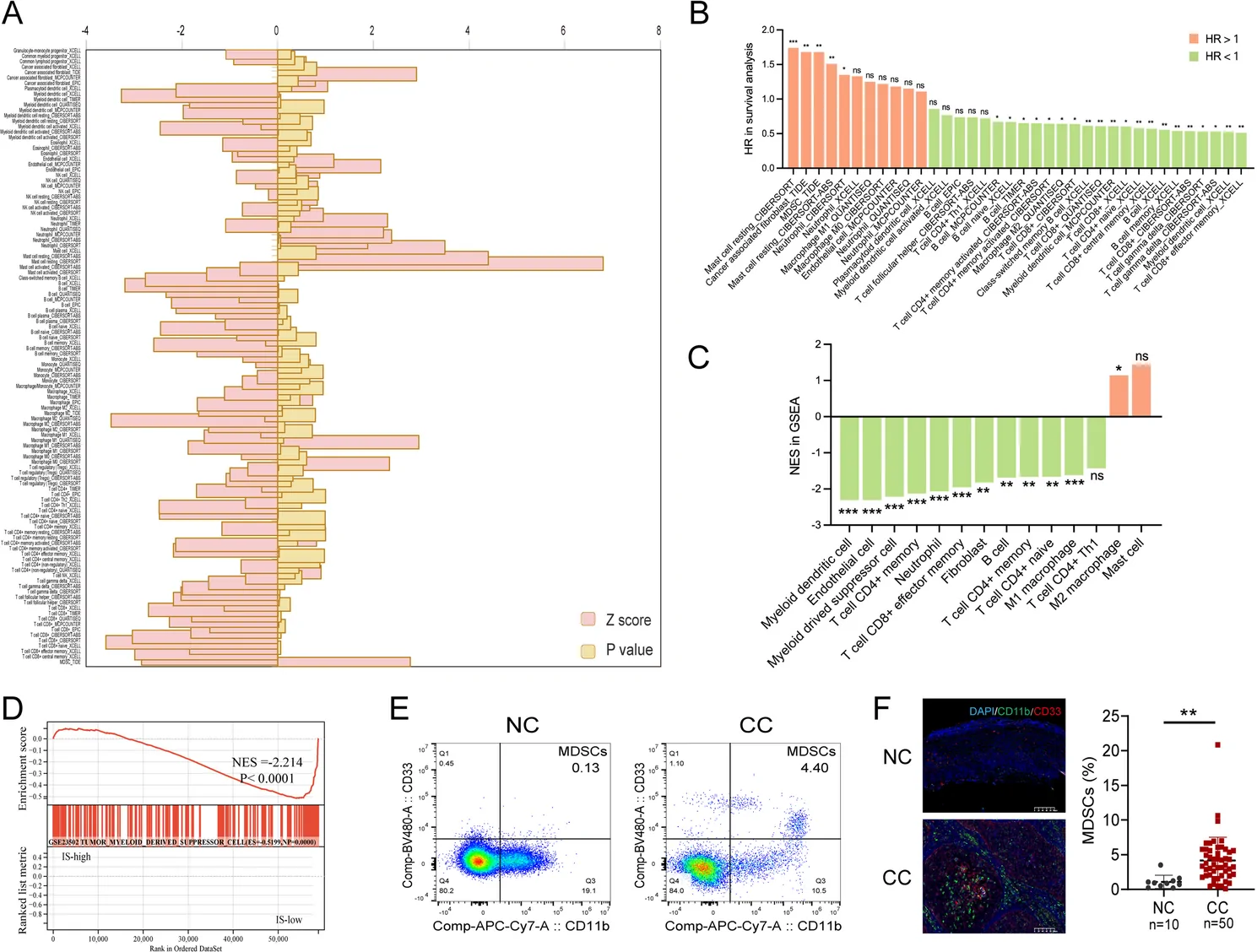
MDSCs was identified as key components by the tumor immune microenvironment analysis, predicting poor survival in CC. (**A**) TCGA-CC immune microenvironment infiltration estimation analysis via Timer 2.0 online datasets. (**B**) Survival analysis based on CC immune microenvironment infiltration in Timer 2.0 online dataset. (**C**) Gene Set Enrichment Analysis of CC immune microenvironment infiltration based on TCGA-ESTIMATE immune scores. Data were presented as normalized enrichment score (NES).(**D**) GESA analysis of MDSCs in high-immunological score group (IS-high) and low-immunological score group (IS-low) groups. NES =-2.214, ES= -0.5199, *P*< 0.0001. (**E**) Flow cytometry analysis of CD33^+^CD11b^+^ MDSCs in the normal cervix (NC) and cervical cancer (CC) tissue samples (NC: *n* = 3; CC: *n*=10). (**F**) Immunohistochemical staining of CD33^+^(red), CD11b^+^(Green), and DAPI (blue) in NC and CC tissue samples (NC: *n* = 10; CC: *n*=50)

To explore the immune microenvironment components change in CC, we first applied TCGA-CC immune microenvironment infiltration estimation analysis via Timer 2.0 online datasets (Fig. 1A). Besides, the survival and the GSEA analyses based on the immune microenvironment infiltration were also executed (Fig. 1B and C, and S1). Combining these 3 analyses, MDSCs infiltration remained one of the most essential processes in the CC immune microenvironment, encouraging us to explore the role of MDSCs in CC further. The GSEA function analysis showed a significant enrichment difference of MDSCs infiltration between high and low immunological score groups (High-IS vs. Low-IS, NES =-2.214, ES= -0.5199, *P* < 0.001, Fig. 1D). What’s more, CD33^+^CD11b^+^ MDSCs were enriched in CC than the normal cervix (NC) tissue samples, (4.4% vs. 0.13% MDSCs in CC and NC, flow cytometry, Fig. 1E; around 2 times increase of MDSCs in CC vs. NC, IF analysis, Fig. 1F). Taken together, we identified MDSCs as the key components in CC immune microenvironment and high MDSCs infiltration predicted poor prognosis in CC patients.

### Tumor-conditioned MDSCs show enhanced proliferation, migration, and iNOS expression in CC

**Fig. 2:**
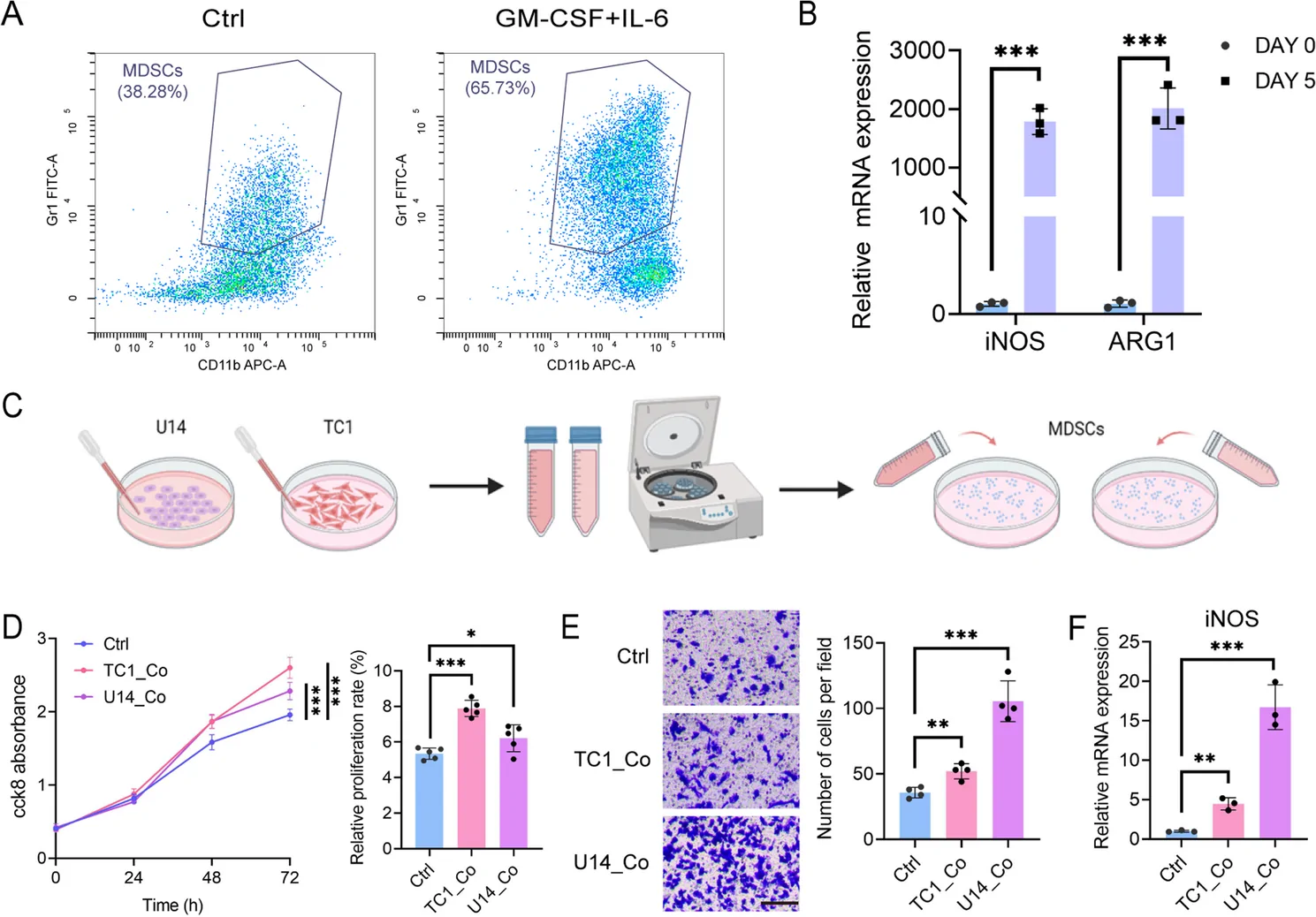
Tumor-conditioned MDSCs show enhanced proliferation, migration, and iNOS expression in CC. **(A)** Flow cytometry analysis of GM-CSF and IL-6 induced Gr1^+^CD11b^+^ MDSCs in vitro from mouse bone marrow (*n* = 3). **(B)** qPCR analysis of MDSCs key functional molecules, iNOS and ARG1 in GM-CSF and IL-6 induced Gr1^+^CD11b^+^ MDSCs in vitro from mouse bone marrow (*n* = 3). **(C)** Diagram of the co-culture system with murine CC cell lines U14, TC1, and MDSCs. The TC1 and U14 cell culture supernatants were added to the MDSCs culture medium to establish the co-culture system. (**D**) MDSCs were cultured in a 1:1 mixture of U14- or TC1-conditioned medium and fresh complete medium. CCK8 proliferation assays of MDSCs treated with Ctrl, TC1_Co, U14_Co (n = 5). Relative proliferation rate was calculated as (OD₇₂ − OD₀) / (OD₀ × 72 h). **(E)** Transwell migration assays of Ctrl-MDSCs, TC1_Co-MDSCs, U14_Co-MDSCs (*n* = 4). **(F)** qPCR analysis of iNOS among Ctrl-MDSCs, TC1_Co-MDSCs, U14_Co-MDSCs (*n* = 3). *P < 0.05; **P < 0.01; ***P < 0.001

In the tumor microenvironment, the interaction between tumor cells and stromal cells plays essential roles in cancer progression [2]. Having established that MDSCs are enriched in CC tissues and associated with poor patient prognosis, we next sought to investigate the functional impact of the tumor microenvironment on MDSCs biology. To this end, we developed an in vitro model using tumor cell-conditioned medium to recapitulate the tumor–MDSCs interaction and to explore the underlying molecular mechanisms. First, we successfully induced murine-MDSCs from bone marrow cells, and the MDSCs key functional molecules, iNOS and ARG1 were also increased along the induction (Fig. 2A and B). Murine CC cell line U14 and TC1 culture supernatants were added into the culture medium of MDSCs to establish the CC-conditioned MDSCs (Fig. 2C). CCK8 assays proved that two CC-conditioned MDSCs, TC1-cocultured MDSCs (TC1_Co) and U14-cocultured MDSCs (U14_Co), had increased proliferation abilities (Fig. 2D). Notably, raised migration abilities were also observed in TC1_Co and U14_Co groups compared to the control (Ctrl) group (Fig. 2E), indicating that CC cells could recruit MDSCs in the TME. Furthermore, qPCR analysis revealed that CC-conditioned MDSCs expressed significantly higher levels of iNOS expression (Fig. 2F).

### Proteomics analyses of tumor-conditioned MDSCs in CC

**Fig. 3:**
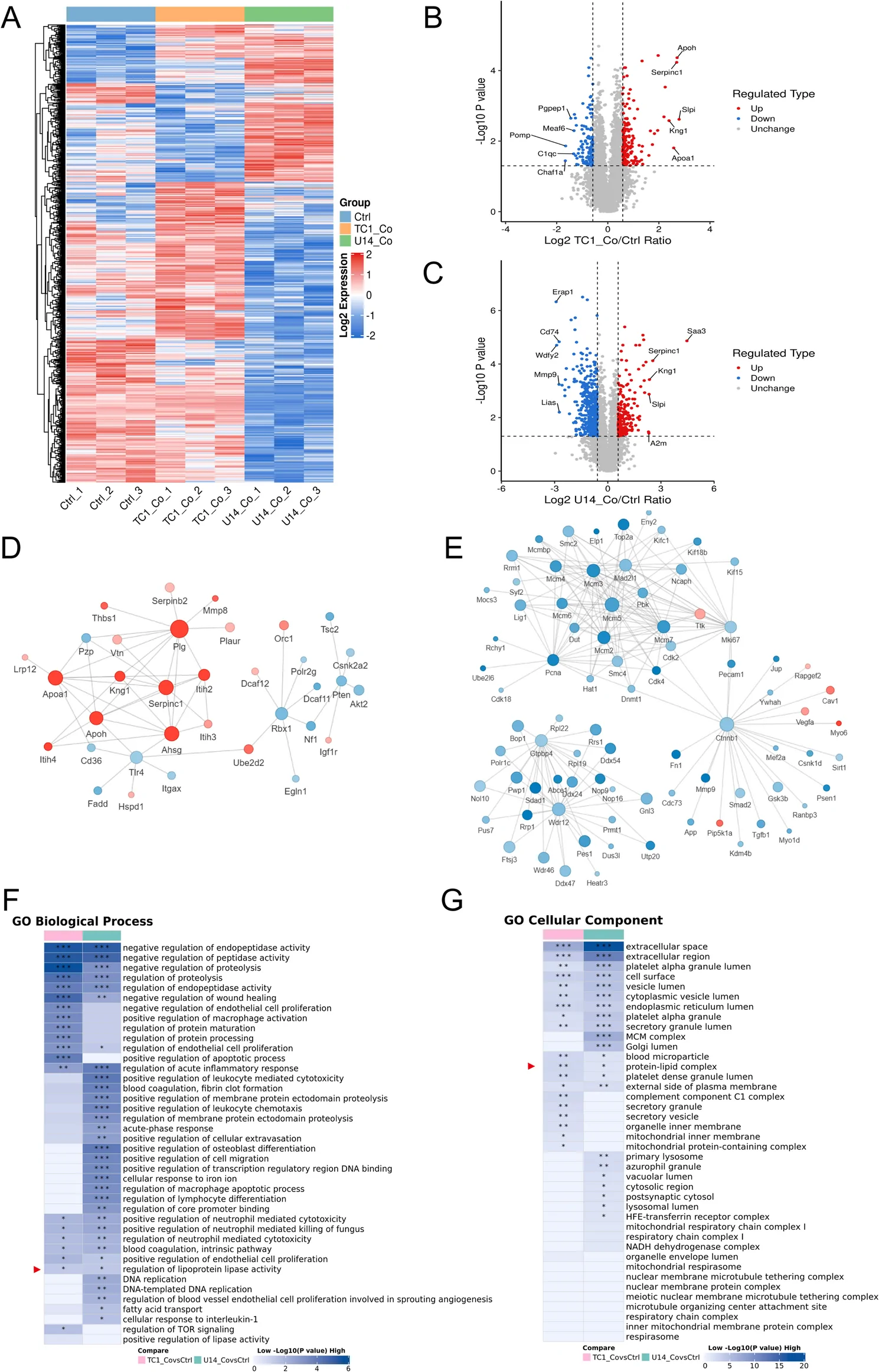
Proteomics analysis of tumor-conditioned MDSCs in CC. (**A**) Heatmap of differentially expressed proteins among MDSCs treated with Ctrl-MDSCs, TC1_Co-MDSCs, U14_Co-MDSCs based on proteomics data (n = 3). (**B**) Volcano plot of DEPs between Ctrl-MDSCs and TC1_Co-MDSCs groups. Blue dots represented downregulated proteins while red dots marked upregulated proteins. The top 5 upregulated and downregulated proteins were marked. (**C**) Volcano plot of DEPs between Ctrl-MDSCs and U14_Co-MDSCs groups. Blue dots represented downregulated proteins while red dots marked upregulated proteins. The top 5 upregulated and downregulated proteins were marked. (**D**) Protein-protein interaction networks based on the top 10 proteins with the highest confidence score (>0.7) in the STRING database. Red marked upregulated genes while blue indicated downregulated genes (TC1_Co-MDSCs vs. Ctrl-MDSCs). (**E**) PPI networks based on the top 10 proteins with the highest confidence score (>0.7) in the STRING database. Red marked upregulated genes while blue indicated downregulated genes (U14_Co-MDSCs vs. Ctrl-MDSCs). (**F**) Gene Ontology biological process function analyses among Ctrl-MDSCs, TC1_Co-MDSCs, and U14_Co-MDSCs groups. The red arrow points to lipid metabolism-related pathways. (**G**) GO cellular component function analyses among Ctrl-MDSCs, TC1_Co-MDSCs, and U14_Co-MDSCs groups. The red arrow points to lipid metabolism-related pathways

Recently, several studies implied that metabolism alteration contributed to the function of MDSCs and proteins are the direct executors of vital movement within the cells [9, 10]. In this case, we chose proteomics and metabolomics analyses to investigate the molecular mechanism behind the CC-conditioned MDSCs. Proteomics analyses were first applied in TC1_Co- and U14_Co-MDSCs (Fig. 3A). The top 5 upregulated and downregulated differentially expressed proteins (DEPs) were marked in the volcano plots (Fig. 3B and C), among which Serpinc1, Kng1, and Slpi were commonly altered in both cells. Protein-protein interaction (PPI) networks based on the top 10 proteins with the highest confidence score (> 0.7) were also analyzed and may provide further protein network mechanism investigation (Fig. 3D and E). To inspect the potential function of MDSCs in CC, we conducted the function enrichment analyses based on Gene Ontology (GO) terms (biological process and cellular components), Clusters of Orthologous Groups of proteins Ontology (COG), Kyoto Encyclopedia of Genes and Genomes (KEGG) annotations (Fig. 3F and G and S2). Since metabolism was one of the focuses of our investigation, we paid much attention to metabolism-related process terms. Regulation of lipoprotein lipase activity and protein-lipid complex were commonly enriched in TC1_Co vs. Ctrl and U14_Co vs. Ctrl comparisons (Fig. 3F and G), indicating that lipid metabolism may contribute to the function of MDSCs in CC.

### Metabolomics analyses of tumor-conditioned MDSCs in CC

**Fig. 4:**
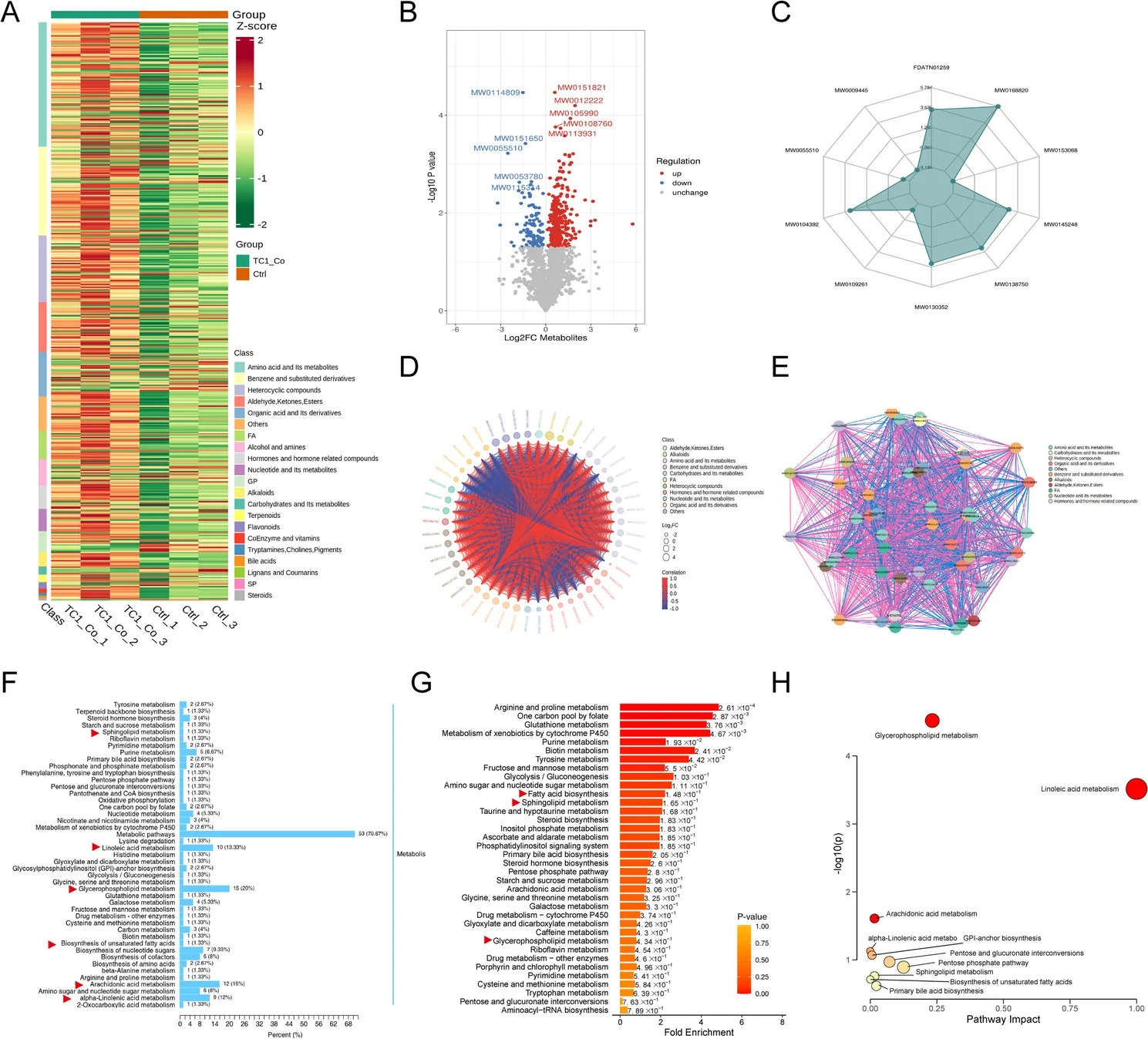
Metabolomics analysis of TC1 CC cell-conditioned MDSCs. (**A**) Heatmap of differentially accumulated metabolites (DAMs) defined by classes among MDSCs treated with normal culture medium (Ctrl), and TC1 cell culture supernatant (TC1_Co) groups based on metabolomics data (*n* = 3). (**B**) Volcano plot of ss between Ctrl-MDSCs and TC1_Co-MDSCs groups. Blue dots represented downregulated metabolites while red dots marked upregulated metabolites. The top 5 upregulated and downregulated metabolites were marked. (**C**) Radar plot of DAMs with top 10 highest absolute value of log_2_FC between Ctrl-MDSCs and TC1_Co-MDSCs groups. (**D**) The chord diagram of DAMs with the top 50 highest variable importance in projection (VIP) values based on the Pearson correlation indexes between Ctrl-MDSCs and TC1_Co-MDSCs groups showed the metabolic proximities among those DAMs. (**E**) The metabolic proximity network of DAMs with the top 50 highest VIP values between the Ctrl-MDSCs and TC1_Co-MDSCs groups. (**F**) Kyoto Encyclopedia of Genes and Genomes metabolism function analysis of DAMs between the Ctrl-MDSCs and TC1_Co-MDSCs groups. The red arrows pointed to lipid metabolism-related pathways. (**G**) Metabolite set enrichment analysis showed enriched pathways between the Ctrl-MDSCs and TC1_Co-MDSCs groups. The red arrows pointed to lipid metabolism-related pathways. (**H**) The lipid metabolism-related pathway analysis based on the metabolomics data

Subsequently, metabolomics analyses were executed in TC1_Co- and Ctrl-MDSCs, the results of which were clustered based on metabolite classes in the heat map picture (Fig. 4A). And the top 5 upregulated and downregulated differentially accumulated metabolites (DAMs) were marked (Fig. 4B and C). The chord diagram and proximity network of DAMs with the top 50 highest variable importance in projection (VIP) values based on the Pearson correlation indexes between Ctrl-MDSCs and TC1_Co-MDSCs groups were analyzed to explore the correlation between the DAMs (Fig. 4D and E, and S3). Kyoto Encyclopedia of Genes and Genomes (KEGG) metabolism function analysis, metabolite set enrichment analysis (MSEA), and the lipid metabolism-related pathway enrichment analysis of DAMs between these groups showed that lipid metabolism-related pathways were enriched, including sphingolipid metabolism, linoleic acid metabolism, glycerophospholipid metabolism, fatty acid biosynthesis, and arachidonic acid metabolism (Fig. 4F and H). Among these analyses, sphingolipid metabolism, fatty acid biosynthesis, and glycerophospholipid metabolism were the common enriched pathways in all 3 analyses. The same metabolomics analyses were applied in U14_Co- and Ctrl-MDSCs (Fig S4A-H, and S5). Among the metabolism enrichment analyses, sphingolipid metabolism, fatty acid biosynthesis, arachidonic acid metabolism, and glycerophospholipid metabolism were commonly enriched (Fig S4F-H). Combining the results of metabolomics analyses in TC1_Co and U14_Co co-culture systems, sphingolipid metabolism, fatty acid biosynthesis, and glycerophospholipid metabolism stood out as shared altered lipid metabolism pathways.

### Integrated analysis of the proteomics and metabolomics profiling of tumor-conditioned MDSCs in CC

**Fig. 5:**
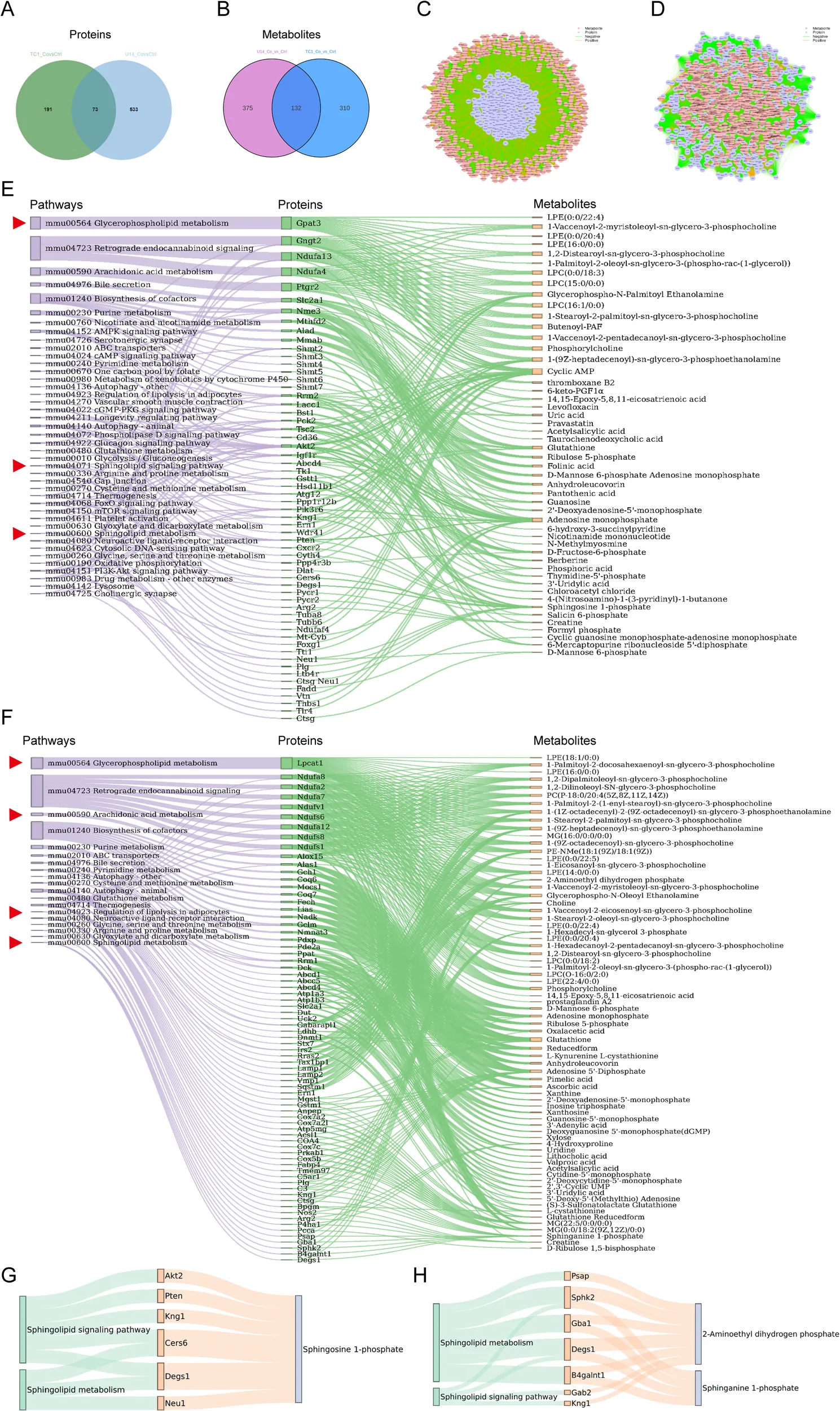
Integrated analysis of proteomics and metabolomics reveals sphingolipid metabolism alteration in TC1/U14 CC cell-conditioned MDSCs. (**A**) Venn diagram of overlapping differentially expressed proteins (DEPs) among MDSCs treated with Ctrl-MDSCs, TC1_Co-MDSCs, U14_Co-MDSCs (*n* = 3). (**B**) Venn diagram of overlapping differentially accumulated metabolites (DAMs) among the Ctrl-MDSCs, TC1_Co-MDSCs, and U14_Co-MDSCs groups. (**C**) Protein–metabolite interaction network between the Ctrl-MDSCs and TC1_Co-MDSCs groups. (**D**) Protein–metabolite interaction network between the Ctrl-MDSCs and U14_Co-MDSCs groups.Protein–metabolite interaction network between the Ctrl-MDSCs and U14_Co-MDSCs groups. (**E**) Sankey diagram of enriched pathways, DEPs, DAMs between the Ctrl-MDSCs and TC1_Co-MDSCs groups. The red arrows pointed to lipid metabolism-related pathways. (**F**) Sankey diagram of enriched pathways, DEPs, DAMs between the Ctrl-MDSCs and U14_Co-MDSCs groups. The red arrows pointed to lipid metabolism-related pathways. (**G**) Sankey diagram of sphingolipid metabolism pathways, DEPs, DAMs between the Ctrl-MDSCs and TC1_Co-MDSCs groups. (**H**) Sankey diagram of sphingolipid metabolism pathways, DEPs, DAMs between the Ctrl-MDSCs and U14_Co-MDSCs groups

In the above experiments, we have identified Serpinc1, Kng1, and Slpi as shared DEPs in proteomics analyses and sphingolipid metabolism, fatty acid biosynthesis, and glycerophospholipid metabolism as commonly altered pathways in both co-culture systems. Then we aimed to execute the integrated analysis to establish the protein–metabolite network. First, 73 common proteins and 132 metabolites were screened in both comparisons (Fig. 5A and B). And the protein–metabolite networks were established to show the interaction between DEPs and DAMs in these co-culture systems (Fig. 5C and D). Moreover, the enriched pathway protein–metabolite networks were listed in Sankey diagrams (Fig. 5E and F). In the TC1_Co co-culture system, glycerophospholipid metabolism, sphingolipid signaling pathway, and sphingolipid metabolism were enriched (Fig. 5E), while in the U14_Co system, glycerophospholipid metabolism, arachidonic acid metabolism, regulation of lipolysis in adipocytes, and sphingolipid metabolism were identified (Fig. 5F). Notably, glycerophospholipid metabolism and sphingolipid metabolism were commonly enriched in both co-culture systems. However, integrated protein–metabolite network analysis revealed that no shared differentially expressed proteins could be linked to glycerophospholipid metabolism across the two models, making it difficult to pinpoint a specific regulatory node for further mechanistic investigation. In contrast, sphingolipid metabolism not only exhibited shared protein changes across both systems, but these proteins consistently converged on the same downstream metabolite—sphingosine-1-phosphate (S1P) (Fig. 5G and H). This convergence provided a clear and tractable protein–metabolite regulatory node for functional validation. Therefore, we prioritized sphingolipid metabolism, with a particular focus on S1P, for subsequent mechanistic studies.

### Kng1–S1P axis was identified as key protein–metabolite network

**Fig. 6:**
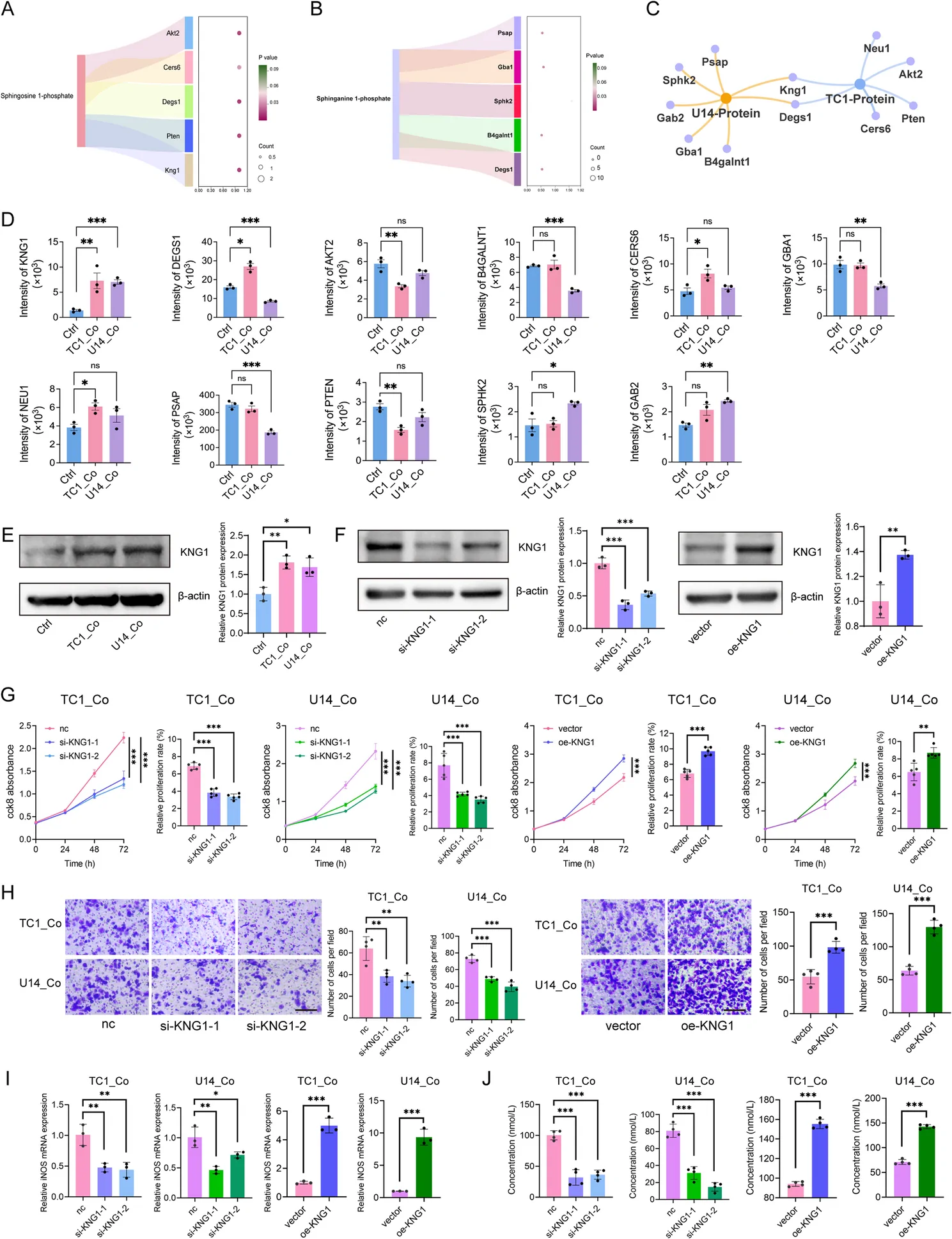
Kng1-mediated sphingolipid metabolism was defined as a potential essential process in TC1/U14 CC cell-conditioned MDSCs. (**A**) Sankey and bubble diagram of DEPs and DAMs in sphingolipid metabolism between the Ctrl-MDSCs and TC1_Co-MDSCs groups. (**B**) Sankey and bubble diagram of DEPs and DAMs in sphingolipid metabolism between the Ctrl-MDSCs and U14_Co-MDSCs groups. (**C**) Metabolite–protein interaction network in sphingolipid metabolism based on DEPs and DAMs of Ctrl-MDSCs, TC1_Co-MDSCs, and U14_Co-MDSCs groups. (**D**) The protein expression level of DEPs in sphingolipid metabolism among Ctrl-MDSCs, TC1_Co-MDSCs, and U14_Co-MDSCs groups. (**E**) Western blot analysis of Kng1 protein expression in Ctrl-MDSCs, TC1_Co-MDSCs, and U14_Co-MDSCs groups. β-actin was used as a loading control. The bar chart shows the relative Kng1 protein levels normalized to β-actin. (**F**) Western blot analysis confirming the efficiency of KNG1 knockdown (si-KNG1) and overexpression (oe-KNG1) in MDSCs. β-Actin was used as a loading control. (**G**) Cell proliferation was assessed by CCK-8 assay in TC1_Co- and U14_Co-conditioned MDSCs following KNG1 knockdown or overexpression (*n* = 5). (**H**) Transwell migration assays of TC1_Co- and U14_Co-conditioned MDSCs after KNG1 knockdown or overexpression. Representative images and quantitative analysis are shown (*n* = 4). (**I**) qPCR analysis of iNOS mRNA levels in TC1_Co- and U14_Co-conditioned MDSCs after modulation of KNG1 expression. Data were normalized to β-actin (*n* = 3). (**J**) Concentration of sphingosine 1-phosphate in the culture medium of TC1_Co- and U14_Co-conditioned MDSCs was measured by ELISA following KNG1 knockdown or overexpression (*n* = 4). *P < 0.05; **P < 0.01; ***P < 0.001; ns = not significant

We next examined the protein–metabolite interaction networks within the sphingolipid pathway to identify candidate regulators (Fig. 6A and C). In both co-culture systems, sphingolipid metabolism converged on S1P through multiple protein connections, including AKT2, Cers6, Degs1, Pten, Kng1 in TC1_Co, and Sphk2, Degs1, Psap, Gba1 in U14_Co. To further validate the expression level of these DEPs, the expression patterns in proteomics were analyzed and only Kng1 was upregulated in TC-1_Co and U14_Co groups than the control group (Fig. 6D and E).

**Fig. 7:**
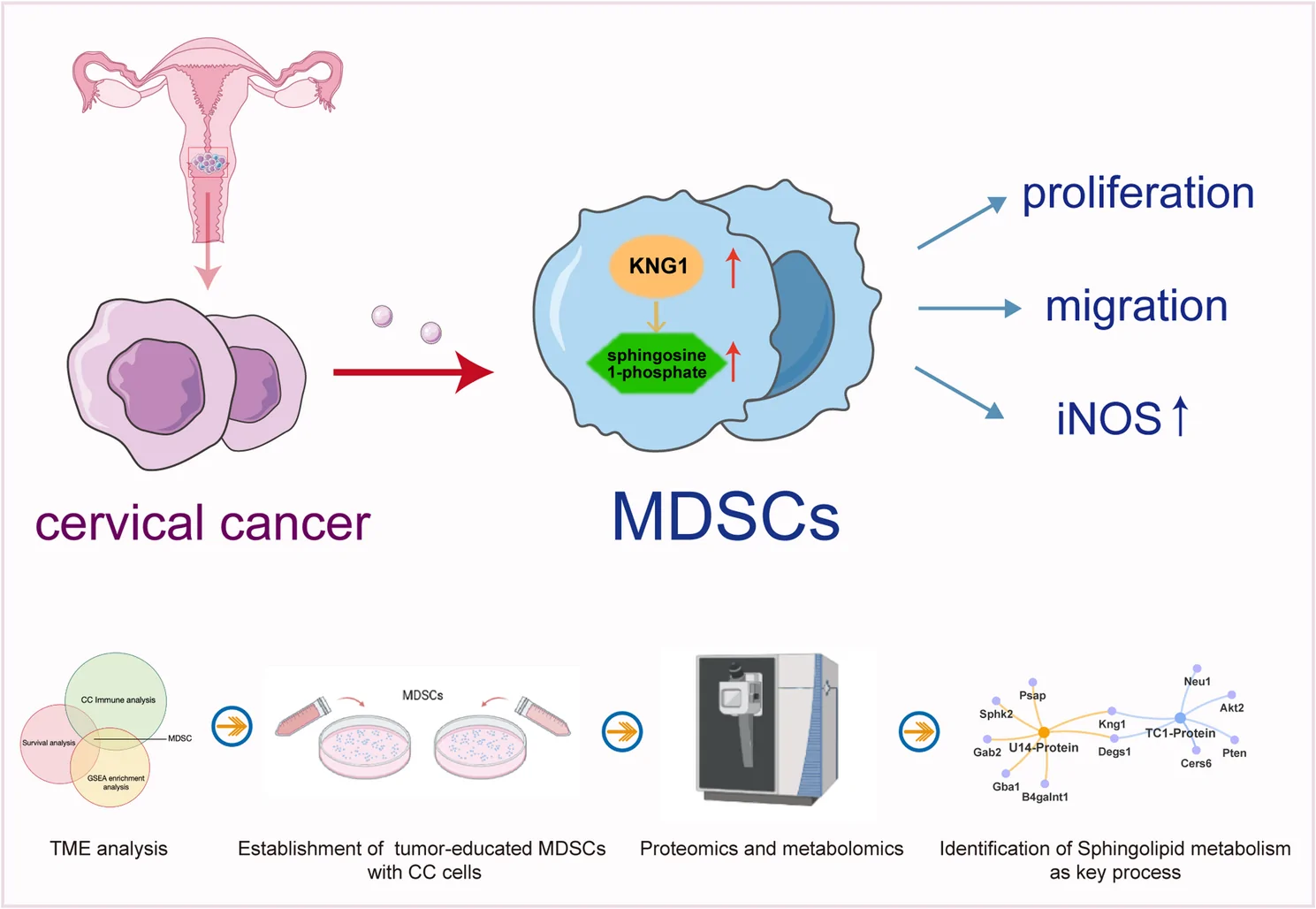
Schematic overview of the study workflow and key findings. This schematic summarizes the integrated multi omics approach. Tumor immune infiltration analysis of TCGA CC datasets identified MDSCs as a poor prognosis component. Mouse cervical cancer conditioned MDSCs were generated using culture supernatants from U14 and TC1 cell lines and exhibited enhanced proliferation, migration and iNOS expression. Proteomics and metabolomics revealed prominent sphingolipid metabolic reprogramming, and integrated network analysis and gain and loss of function experiments pinpointed the Kng1–sphingosine-1 phosphate axis as a central regulatory node

To validate the functional role of the Kng1–S1P axis in CC-conditioned MDSCs, we modulated Kng1 expression using siRNA-mediated knockdown and plasmid-induced overexpression (Fig. 6F). CCK-8 assays demonstrated that knockdown of KNG1 significantly attenuated the proliferation of two CC-conditioned MDSC lines, TC1_Co and U14_Co. Conversely, KNG1 overexpression enhanced their proliferative capacity (Fig. 6G). Similarly, KNG1 depletion led to reduced migratory ability and decreased production of iNOS. These functional impairments were rescued by KNG1 overexpression (Fig. 6H and I). Collectively, these results indicate that CC cells regulate the proliferation, migration, and immunosuppressive function of MDSCs within TME by modulating KNG1 expression. Furthermore, knockdown of KNG1 resulted in a concomitant decrease in S1P levels (Fig. 6J). Integrating the interaction network data with our functional validation, we propose that the Kng1–S1P axis constitutes a key protein–metabolite regulatory network in CC cell-conditioned MDSCs (Fig. 7).

## Discussion

The prognosis of CC is critically linked to disease stage, with advanced or metastatic cases exhibiting a 5-year survival rate below 20%. Current standard therapies, including surgery, radiotherapy, and chemotherapy, often provide limited efficacy. Consequently, immunotherapy has emerged as a promising strategy, particularly for recurrent or metastatic disease [3]. Extensive research has employed bioinformatics analysis, single-cell transcriptomics, and immunofluorescence approaches to characterize the CC immune microenvironment and identify key cellular subsets involved in immune regulation [11–13]. Predictive models for immunotherapy response are also under development [14, 15].

However, immunotherapy outcomes in CC remain suboptimal, largely due to the immunosuppressive TME [16]. MDSCs are key immunosuppressive players associated with poor prognosis in multiple cancers, including but not limited to breast cancer, prostate cancer, and pancreatic cancer [17–19]. In CC, elevated levels of circulating MDSCs and their secreted factors including iNOS, IDO, and ARG1 have been reported [7, 20, 21]. Our analysis further confirms that high MDSCs infiltration in CC tumors correlates with poor prognosis. Tumor cells are known to remodel the TME by educating immune cells to support immune evasion [22–25]. Aligning with this, we demonstrate that CC cells can directly educate MDSCs, enhancing their proliferative and migratory capacities.

Metabolic reprogramming is essential for MDSCs to adapt to and function within the stressful TME [9, 26]. While they utilize enhanced glycolysis, fatty acid oxidation, and glutaminolysis to fuel immunosuppressive activities [27, 28], emerging evidence highlights a pivotal role for lipid metabolism. Tumor-derived signals G-CSF and GM-CSF can upregulate lipid transport receptors in MDSCs, boosting exogenous lipid uptake [29]. Key lipid-associated molecules, such as free fatty acid receptor 2 in lung adenocarcinoma MDSCs, link lipid sensing to immunosuppressive effector functions and correlate with poor prognosis [30]. In polymorphonuclear MDSCs (PMN-MDSCs), upregulated receptors for oxidized low-density lipoprotein and fatty acid transport protein 2 facilitate their differentiation and enhance the synthesis of immunosuppressive mediators like prostaglandin E2 [31, 32]. Furthermore, fatty acid uptake activates fatty acid oxidation in MDSCs, and inhibiting FAO can reverse their immunosuppression and synergize with chemotherapy [33]. Consistently, our proteomic and metabolomic analyses identified significant enrichment of lipid-related processes—including lipoprotein lipase activity regulation, sphingolipid metabolism, fatty acid biosynthesis, and glycerophospholipid metabolism—in CC-conditioned MDSCs. These findings underscore lipid metabolic reprogramming as a central mechanism in MDSCs function and suggest its potential as a therapeutic target.

Sphingolipid metabolism is increasingly recognized as a critical regulator in cancer progression and immune modulation [34]. Within TME, the availability of substrates like serine and palmitic acid can steer sphingolipid synthesis towards fostering an immunosuppressive state, such as promoting Tregs accumulation [35]. Multi-omics studies have further linked sphingolipid metabolism-related genes to immune cell infiltration and tumor progression in cancers like gastric carcinoma [36]. Notably, sphingosine-1-phosphate (S1P) has been increasingly recognized as a key mediator of MDSCs function. Mechanistically, intracellular S1P directly binds and inhibits acetyl-CoA carboxylase 1, rewiring fatty acid metabolism to sustain the immunosuppressive phenotype of MDSCs. In preclinical models of breast cancer, bladder cancer and melanoma that are resistant to checkpoint inhibition therapy, inhibiting SphK2 with drugs can alleviate the immunosuppression mediated by MDSCs, limit tumor progression, and enhance the efficacy of anti-PD-1 treatment [37]. Beyond its intracellular role, S1P may acts extracellularly through its receptor S1PR1 to activate STAT3 signaling, promoting MDSCs recruitment into the tumor microenvironment and facilitating premetastatic niche formation [38]. Interestingly, the S1P receptor modulator FTY720, an FDA-approved immunosuppressant, has been reported to promote tumor growth by triggering MDSCs accumulation via S1PR3-mediated GM-CSF release through Rho kinase and ERK-dependent pathways [39]. Collectively, these studies position S1P signaling as a central regulator of MDSCs-mediated immune suppression. In line with this emerging paradigm, our integrated proteomic and metabolomic analyses reveal that sphingolipid metabolism is profoundly reprogrammed in CC-conditioned MDSCs, with S1P identified as a key downstream effector metabolite.

Beyond its classic role in coagulation, kininogen-1 (Kng1) has been implicated in the progression of multiple cancers, including colorectal cancer, cholangiocarcinoma, glioma, and ovarian cancer [40–44]. Evidence also suggests its involvement in immune regulation, as Kng1 deficiency can attenuate inflammatory cytokine production and immune cell infiltration in models such as inflammatory bowel disease [45]. Here, we uncover a novel function for Kng1 within the tumor immune microenvironment. We demonstrate that Kng1 plays a critical role in modulating the biological behavior and immunosuppressive function of CC-conditioned MDSCs through the regulation of sphingolipid metabolism. Functional studies via Kng1 knockdown and overexpression confirmed its necessity for key processes in these MDSCs, including proliferation, migration, iNOS expression, and S1P production.

Several limitations of this study should be acknowledged. First, our in vitro model utilized conditioned media from murine CC cell lines to generate tumor-conditioned MDSCs, which, while enabling mechanistic dissection, does not fully recapitulate the complex cellular interactions and three-dimensional architecture of the intact tumor microenvironment. Future studies using orthotopic tumor models or patient-derived samples will be necessary to validate these findings in a more physiologically relevant context. Second, although we identified Kng1 as a key node in the protein–metabolite network, the precise molecular mechanism by which Kng1 regulates S1P production—whether through direct interaction with sphingosine kinases, modulation of substrate availability, or indirect signaling cascades—remains to be elucidated.

In summary, by establishing an in vitro model of CC-conditioned MDSCs and employing integrated proteomic and metabolomic profiling, we have identified MDSCs as key poor-prognosis-associated components in CC and revealed their profound lipid metabolic reprogramming, particularly in sphingolipid pathways. Central to this reprogramming is the Kng1–S1P axis, which we define as a critical protein–metabolite regulatory network. Our findings provide novel mechanistic insights into MDSCs-mediated immunosuppression in CC and nominate the Kng1-sphingolipid axis as a potential therapeutic target, warranting future validation in human contexts and exploration of its clinical translatability.

## Supplementary Information

Supplementary Material 1.

Supplementary Material 2.

## Data Availability

The datasets used and/or analysed during the current study are available from the corresponding author on reasonable request.

## Abbreviations

CC: Cervical cancer
NC: Normal cervix
TME: Tumor microenvironment
MDSCs: Myeloid-derived suppressor cells
KEGG: Kyoto Encyclopedia of Genes and Genomes
GO: Gene Ontology
TC1_Co MDSCs: TC1 cell-cocultured myeloid-derived suppressor cells
U14_Co MDSCs: U14 cell-cocultured myeloid-derived suppressor cells
GSEA: Gene Set Enrichment Analysis
S1P: Sphingosine-1-phosphate
