# Supplementary material for: Multi-omics profiling reveals sphingolipid metabolism reprogramming of tumor-conditioned MDSCs in cervical cancer
Source: BMC Cancer. 2026 Sep 18;26:1122. doi: 10.1186/s12885-026-16914-2 (PMC13589348; doi:10.1186/s12885-026-16914-2)

Fig7E KNG1

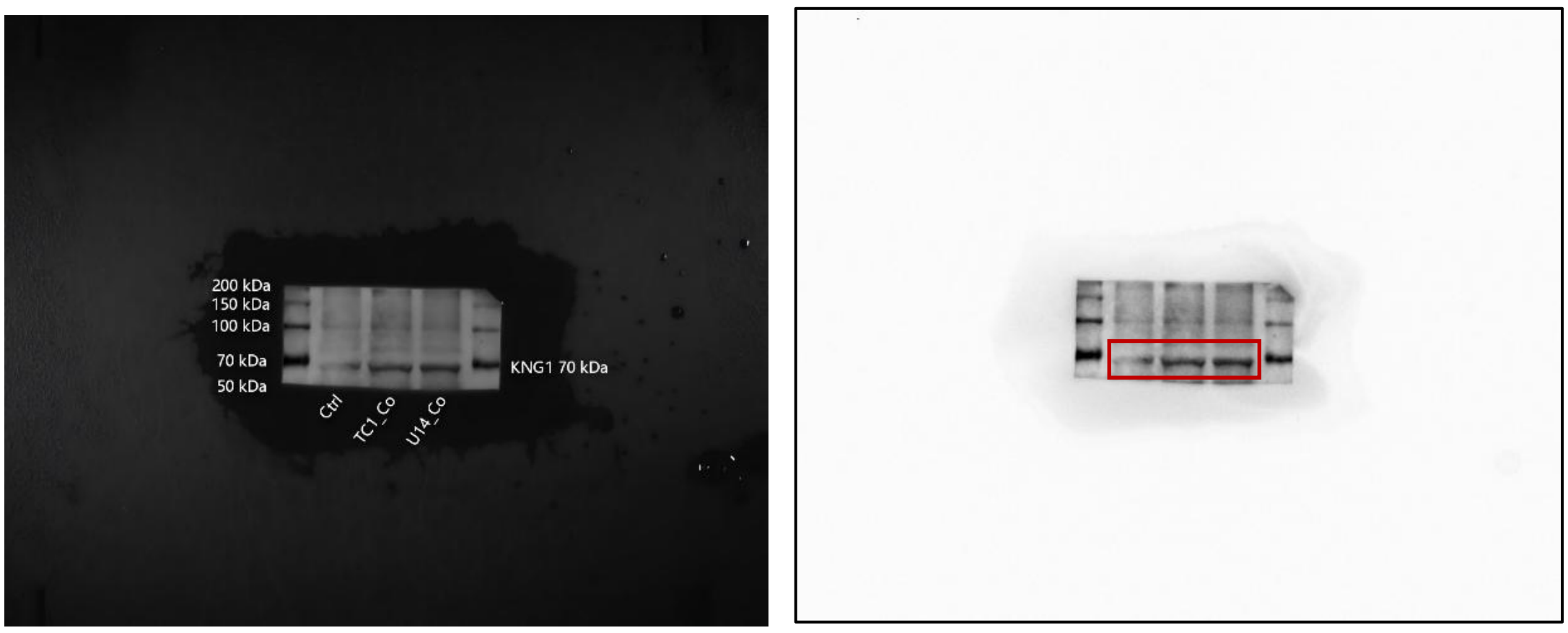

Fig7E β-actin

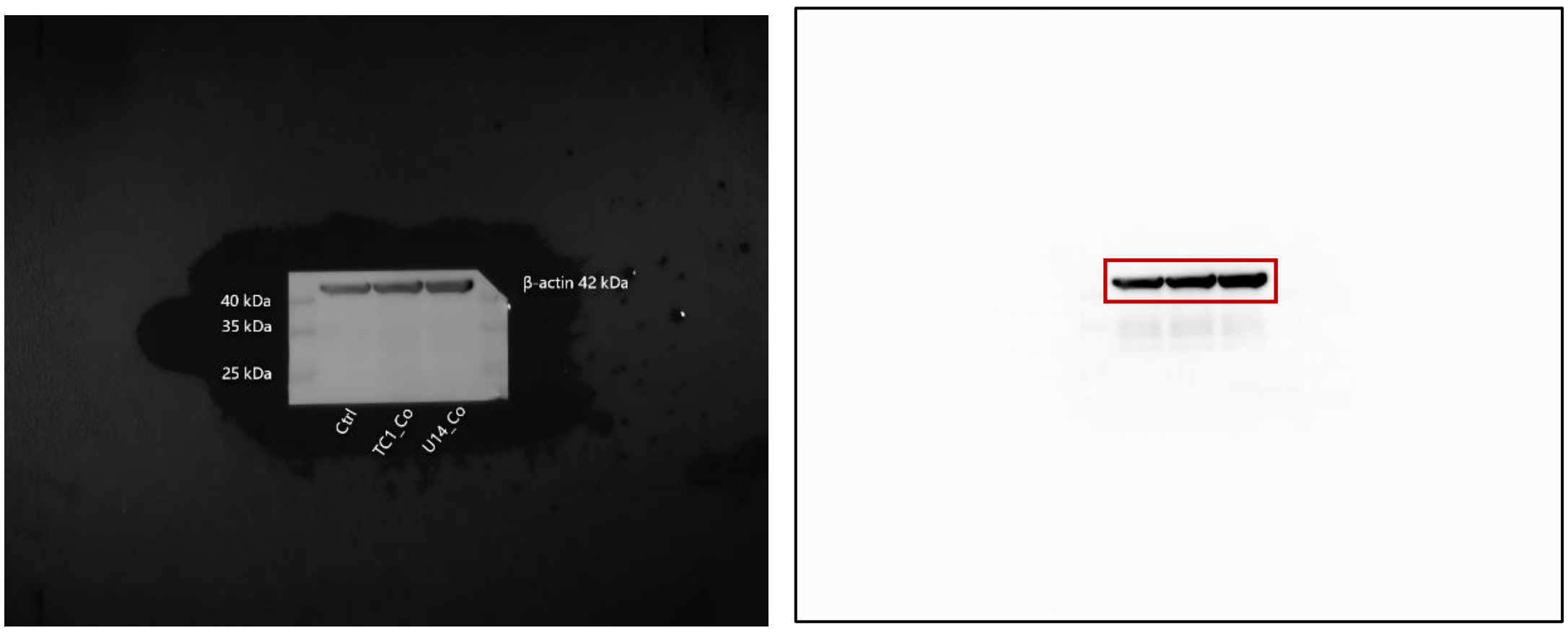

Fig7F si-KNG1

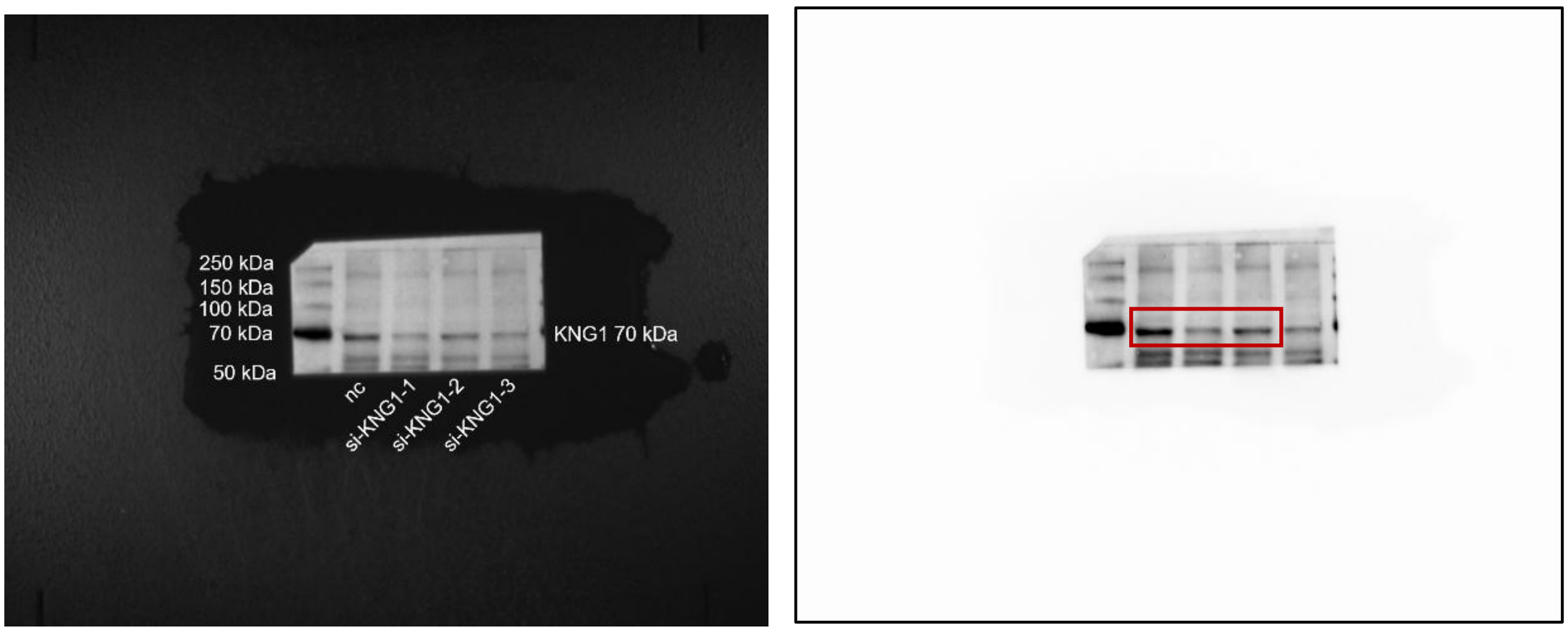

Fig7F si-β-actin

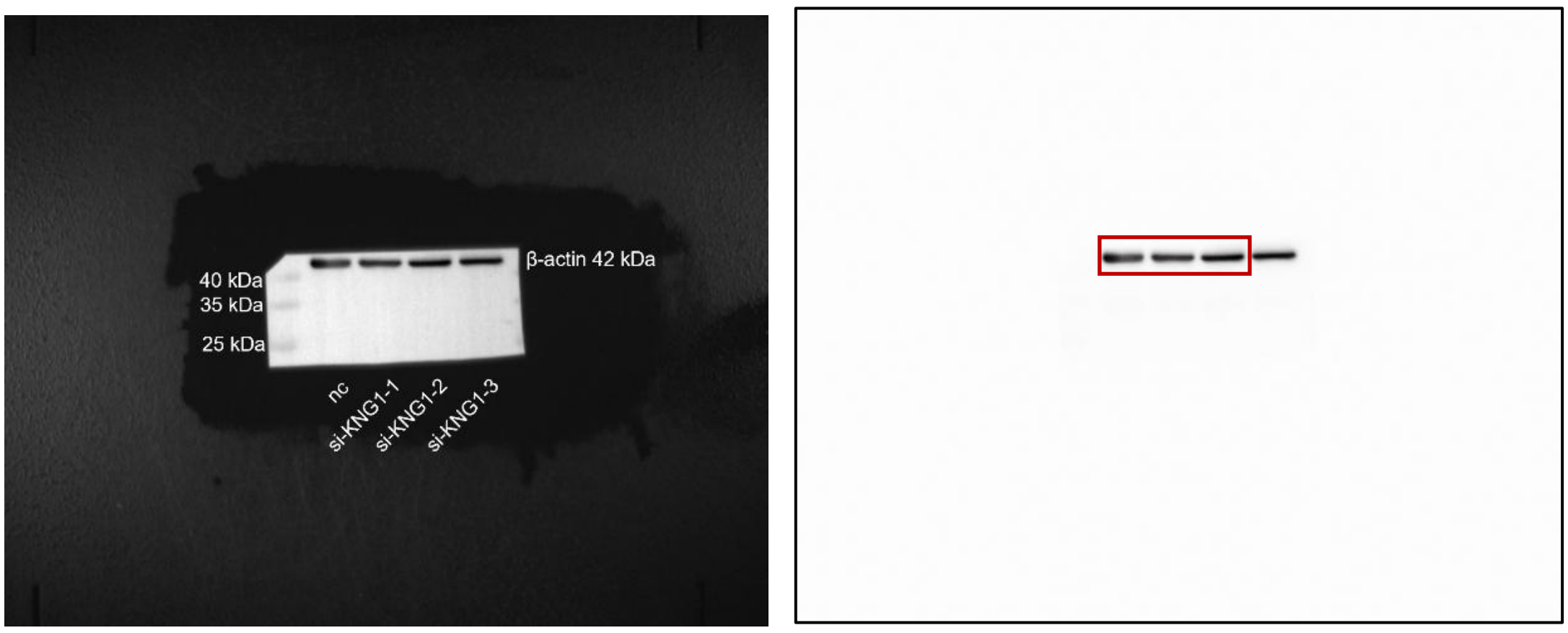

Fig7F oe-KNG1

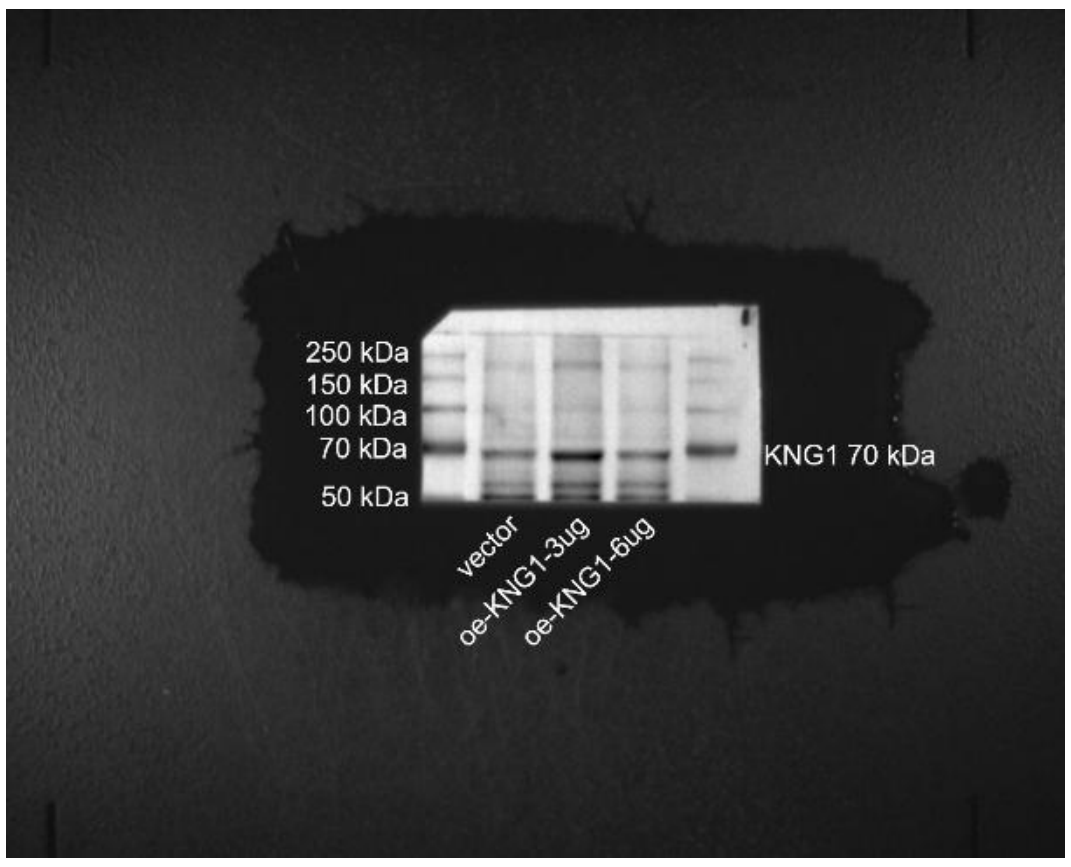

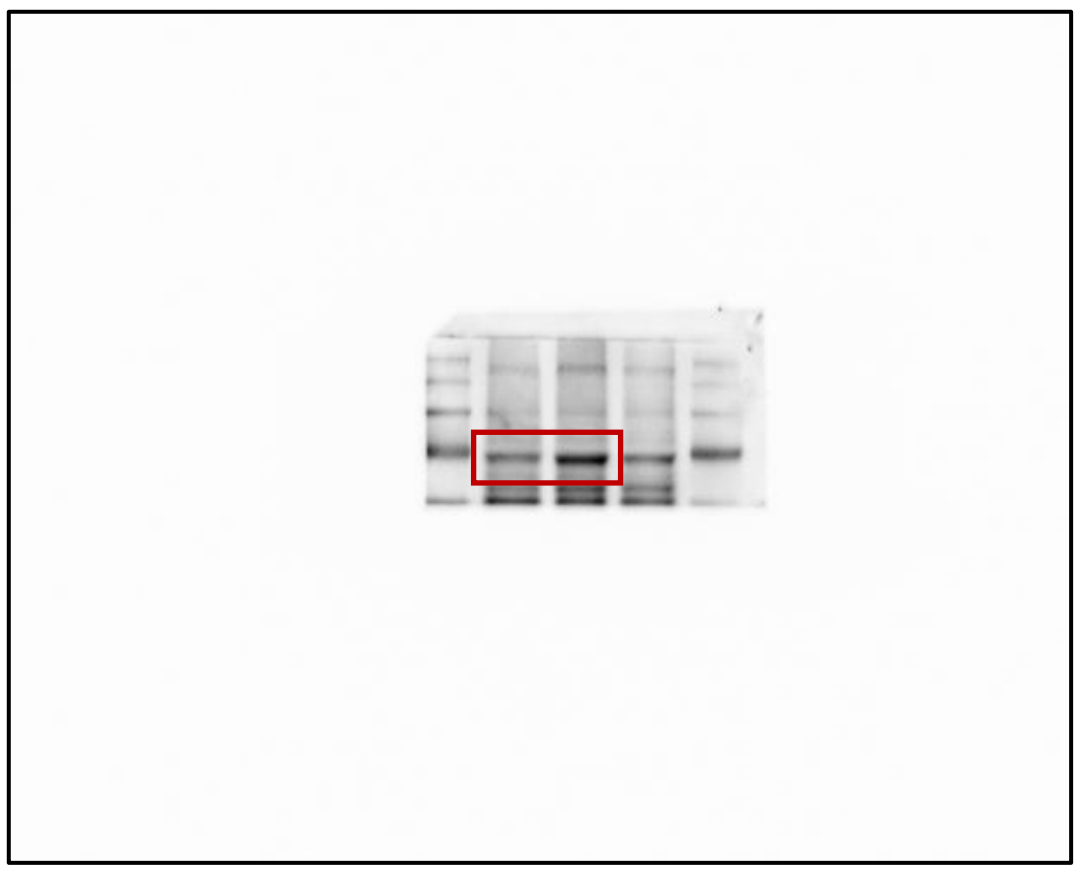

Fig7F oe-β-actin

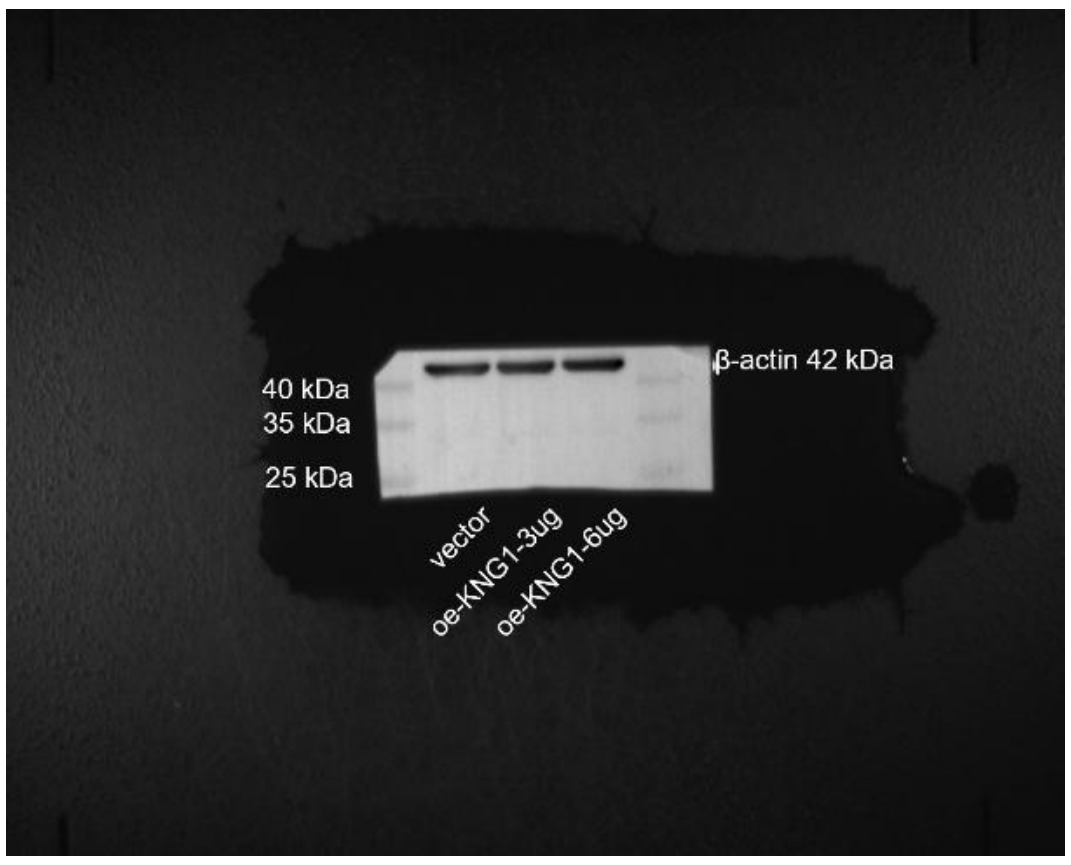

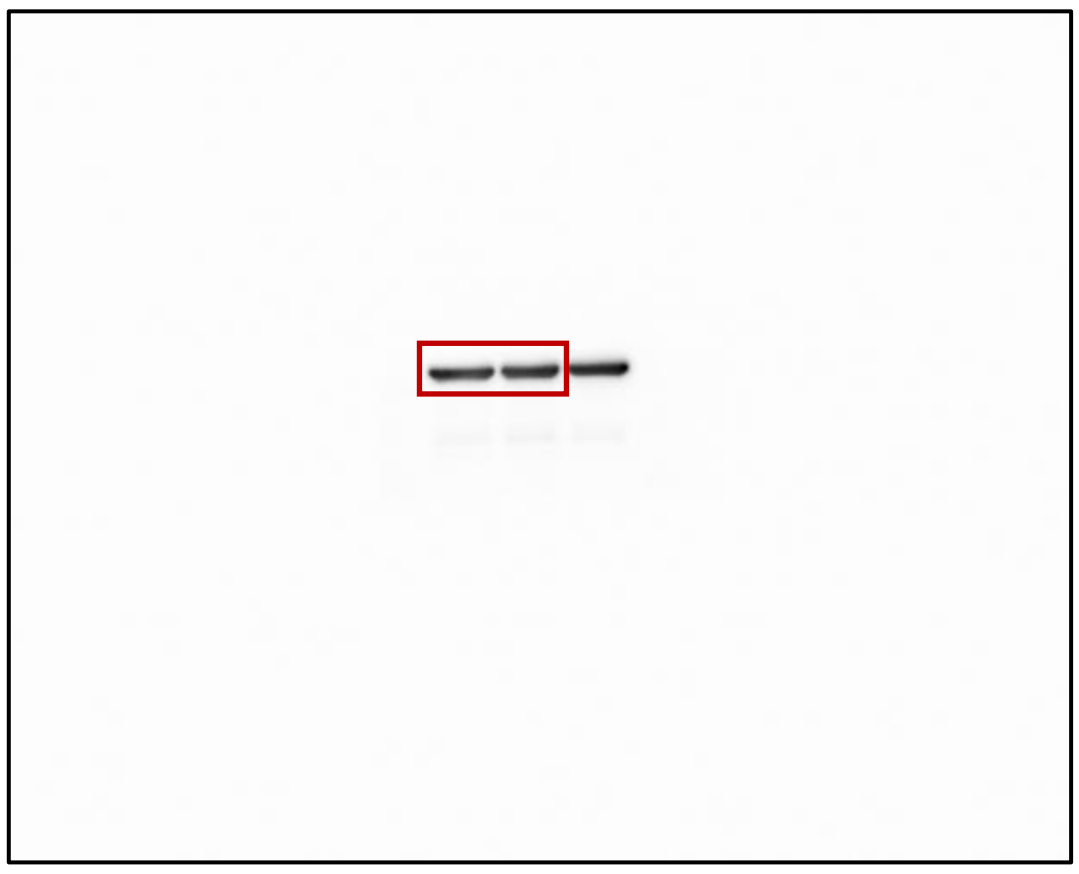

Supplement: Supplementary file 1 [file 12885_2026_16914_MOESM2_ESM.pdf]
