# Supplementary material for: Multi-omics profiling reveals sphingolipid metabolism reprogramming of tumor-conditioned MDSCs in cervical cancer
Source: BMC Cancer. 2026 Sep 18;26:1122. doi: 10.1186/s12885-026-16914-2 (PMC13589348; doi:10.1186/s12885-026-16914-2)
Supplement: Supplementary file 2 [file 12885_2026_16914_MOESM1_ESM.docx]

**Supplementary Information**

**Multi-omics Profiling Reveals Sphingolipid Metabolism Reprogramming of Tumor-conditioned Myeloid-Derived Suppressor Cells in Cervical Cancer**

**This supplementary file includes**:

1. Figures and figure legends for S1 to S5

2. Supplementary Table 1

**Figure S1. Overall survival analysis based on TME components’ scores in CC patients in Timer2.0 Dataset.**

**(A) – (AI)** Overall survival (OS) analysis in CC patients in Timer2.0 Dataset, based on TME components’ scores relatively as B cell memory_XCELL, B cell naive_XCELL, B cell_EPIC, B cell_MCPCOUNTER, B cell_TIMER, B cell_XCELL, Cancer associated fibroblast_TIDE, Class−switched memory B cell_XCELL, Endothelial cell_MCPCOUNTER, Macrophage M0_CIBERSORT, Macrophage M1_QUANTISEQ, Macrophage M2_QUANTISEQ, Mast cell resting_CIBERSORT, Mast cell resting_CIBERSORT−ABS, Myeloid dendritic cell activated_XCELL, Myeloid dendritic cell_MCPCOUNTER, Myeloid dendritic cell_XCELL, Neutrophil_CIBERSORT, Neutrophil_MCPCOUNTER, Neutrophil_QUANTISEQ, Neutrophil_XCELL, Plasmacytoid dendritic cell_XCELL, T cell CD4+ memory activated_CIBERSORT, T cell CD4+ memory activated_CIBERSORT−ABS, T cell CD4+ naive_XCELL, T cell CD4+ Th1_XCELL, T cell CD8+ central memory_XCELL, T cell CD8+ effector memory_XCELL, T cell CD8+_CIBERSORT, T cell CD8+_CIBERSORT−ABS, T cell CD8+_QUANTISEQ, T cell CD8+_XCELL, T cell follicular helper_CIBERSORT−ABS, T cell gamma delta_CIBERSORT, T cell gamma delta_CIBERSORT−ABS. Hazard Ratio (HR) and *P* values were indicated.

**Figure S2. Classification of differentially expressed proteins based on proteomics analysis.**

(A) Clusters of Orthologous Groups of proteins Ontology (COG) classification of differentially expressed proteins (DEPs) between Ctrl-MDSCs and TC1_Co-MDSCs groups.
(B) Gene Ontology (GO) classification of DEP between Ctrl-MDSCs and TC1_Co-MDSCs groups.
(C) Kyoto Encyclopedia of Genes and Genomes (KEGG) classification of DEPs between Ctrl-MDSCs and TC1_Co-MDSCs groups.
(D) COG classification of DEPs between Ctrl-MDSCs and U14_Co-MDSCs groups.
(E) GO classification of DEPs between Ctrl-MDSCs and U14_Co-MDSCs groups.
(F) KEGG classification of DEPs between Ctrl-MDSCs and U14_Co-MDSCs groups.

**Figure S3. Top 50 differentially accumulated metabolites with the highest Variable Importance in Projection (VIP) values between Ctrl-MDSCs and TC1_Co-MDSCs groups.**

Violin diagrams showed the raw intensity of the top 50 differentially accumulated metabolites (DAMs) with the highest Variable Importance in Projection (VIP) values between Ctrl-MDSCs and TC1_Co-MDSCs groups, based on the OPLS-DA model (biological repetition = 3).

**Figure S4. Metabolomics analysis of U14 CC cell-conditioned MDSCs.**

(A) Heatmap of differentially accumulated metabolites (DAMs) defined by classes among MDSCs treated with normal culture medium (Ctrl), and U14 cell culture supernatant (U14_Co) groups based on metabolomics data (n = 3 samples/group).
(B) Volcano plot of DAMs between Ctrl-MDSCs and U14_Co-MDSCs groups. Blue dots represented downregulated metabolites while red dots marked upregulated metabolites. The top 5 upregulated and downregulated metabolites were marked.
(C) Radar plot of DAMs with top 10 highest absolute value of log_2_FC between Ctrl-MDSCs and U14_Co-MDSCs groups.
(D) The chord diagram of DAMs with the top 50 highest variable importance in projection (VIP) values based on the Pearson correlation indexes between Ctrl-MDSCs and U14_Co-MDSCs groups showed the metabolic proximities among those DAMs.
(E) The metabolic proximity network of DAMs with the top 50 highest VIP values between the Ctrl-MDSCs and U14_Co-MDSCs groups.
(F) Kyoto Encyclopedia of Genes and Genomes (KEGG) metabolism function analysis of DAMs between the Ctrl-MDSCs and U14_Co-MDSCs groups. The red arrows pointed to lipid metabolism-related pathways.
(G) Metabolite set enrichment analysis (MSEA) showed enriched pathways between the Ctrl-MDSCs and U14_Co-MDSCs groups. The red arrows pointed to lipid metabolism-related pathways.
(H) The lipid metabolism-related pathway analysis based on the metabolomics data.

**Figure S5. Top 50 differentially accumulated metabolites with the highest Variable Importance in Projection (VIP) values between Ctrl-MDSCs and U14_Co-MDSCs groups.**

Violin diagrams showed the raw intensity of the top 50 differentially accumulated metabolites (DAMs) with the highest Variable Importance in Projection (VIP) values between Ctrl-MDSCs and U14_Co-MDSCs groups, based on the OPLS-DA model (biological repetition = 3).

**Supplementary Table 1. Primer sequences**

| Gene | Forward primer sequence | Reverse primer sequence |
| --- | --- | --- |
| *Actin* | 5’-CTCTCCCTCACGCCATC-3’ | 5’-ACGCACGATTTCCCTCTC-3’ |
| *Nos2*(iNos) | 5’- ACATCGACCCGTCCACAGTAT-3’ | 5’- CAGAGGGGTAGGCTTGTCTC-3’ |
| *Arg1* | 5’-TGTCCCTAATGACAGCTCCTT-3’ | 5’-GCATCCACCCAAATGACACAT-3’ |
